# Correlates, facilitators and barriers of physical activity among primary care patients with prediabetes in Singapore – a mixed methods approach

**DOI:** 10.1186/s12889-019-7969-5

**Published:** 2020-01-02

**Authors:** Raymond Boon Tar Lim, Wei Keong Wee, Wei Chek For, Jayalakshmy Aarthi Ananthanarayanan, Ying Hua Soh, Lynette Mei Lim Goh, Dede Kam Tyng Tham, Mee Lian Wong

**Affiliations:** 1 Saw Swee Hock School of Public Health, National University of Singapore and National University Health System, Tahir Foundation Building, 12 Science Drive 2, #10-01, Singapore, Singapore city, 117549 Singapore; 2 Health Promotion & Preventive Care, National Healthcare Group Polyclinics, 3 Fusionopolis Link, Nexus@one-north, South Tower, #05-10, Singapore, Singapore city, 138543 Singapore; 3 Clinical Services, National University Polyclinics and National University Health System, 1 Jurong East Street 21, Singapore, Singapore city, 609606 Singapore

**Keywords:** Prediabetes, Physical activity, Primary care, Diabetes prevention, Mixed methods, Public health, Singapore

## Abstract

**Background:**

Primary care patients with prediabetes is a priority group in the clinical, organisational and policy contexts. Engaging in regular physical activity is crucial to prevent diabetes for this group. The objectives of the study were to assess factors associated with meeting the recommendation of at least 150 min of moderate/vigorous physical activity weekly, and to explore facilitators and barriers related to the behaviour among primary care patients with prediabetes in Singapore.

**Methods:**

This was a mixed methods study, consisting of a cross-sectional survey involving 433 participants from 8 polyclinics, and in-depth interviews with 48 of them. Adjusted prevalence ratios (aPR) were obtained by mixed effects Poisson regression model. The socio-ecological model (SEM) was applied, and thematic analysis performed.

**Results:**

The prevalence of meeting the recommendation was 65.8%. This was positively associated with being male (aPR 1.21, 95%CI 1.09–1.34), living in 4–5 room public housing (aPR 1.19, 95%CI 1.07–1.31), living in executive flat/private housing (aPR 1.26, 95%CI 1.06–1.50), having family members/friends to exercise with (aPR 1.57, 95%CI 1.38–1.78); and negatively associated with a personal history of osteoarthritis (aPR 0.75, 95%CI 0.59–0.96), as well as time spent sitting or reclining daily (aPR 0.96, 95%CI 0.94–0.98). The recurrent themes for not meeting the recommendation included lacking companionship from family members/friends, medical conditions hindering physical activity (particularly osteoarthritis), lacking knowledge/skills to exercise properly, “no time” to exercise and barriers pertaining to exercise facilities in the neighbourhood. The recurrent themes for meeting the recommendation included family/peer influence, health/well-being concerns and education by healthcare professionals.

**Conclusions:**

Much more remains to be done to promote physical activity among primary care patients with prediabetes in Singapore. Participants reported facilitators and barriers to physical activity at different levels of the SEM. Apart from the individual and interpersonal levels, practitioners and policy makers need to work together to address the organisational, community and policy barriers to physical activity.

## Background

Prediabetes is a health condition where the blood sugar level is higher than normal but still below the threshold for diabetes [1]. The annual conversion rate from prediabetes to diabetes is reported to be 5–10% [2]. Diabetes is associated with cardiovascular disease-related and all-cause mortality [3]. The economic burden of diabetes is huge. Global expenditure related to diabetes and its complications is projected to rise from US$673 billion in 2015 to US$802 billion in 2040 [4]. Worldwide, the diabetes burden is disproportionately high in Asia where 6 in 10 adults in South Asian cities have either prediabetes or diabetes [5].

Lifestyle modification is the main means to prevent cardiovascular diseases and diabetes in individuals with prediabetes [2]. Other than a healthy diet, regular physical activity is also necessary. There is a 30 to 40% lower risk of developing diabetes in moderately active people compared with those who are sedentary [6]. The American Diabetes Association has recommended that individuals with prediabetes should have at least 150 min of physical activity in a week [7]. This increases muscle capillary density, oxidative capacity, lipid metabolism, and insulin signalling proteins [8], which are associated with enhanced insulin action, even without weight loss [9].

There were at least 3 gaps among studies which had been conducted to investigate the prevalence and correlates of physical inactivity in individuals with prediabetes [10–14]. Firstly, majority of these studies were from the West. The lifestyle behaviour of those in Asia would also be different from the West [10, 11]. For example, 62% of the participants with prediabetes reported insufficient physical activity in Canada [10], while two-thirds of them were inactive in Finland [11]. Since Asia Pacific is the region most affected by prediabetes and diabetes [5], more information is needed from this setting. Secondly, the use of behavioural theories to assess the different influences on physical activity [10, 12, 13] was limited in these studies. Among the few studies that had done so, socio-psychological theories at the individual [11] or interpersonal levels [14] were employed. For example, the FIN-D2D study applied the precaution adoption process model [11]. The participants highlighted having companionship for exercise, knowledge about the type of exercises to perform, and higher self-efficacy as facilitators for them to engage in physical activity [11]. Studies pertaining to the influence of physical activity at higher levels (e.g. organisational/institutional, community or societal/policy) were lacking [10–14]. Besides the intrapersonal level, the socio-ecological model (SEM) considers other spheres of influences ranging from the home, school, work and community environments to the impact of public policy on intrapersonal behaviour [15]. The model has been applied on the elderly [16], adolescents [17] and children [18] to promote physical activity but not in those with prediabetes from the primary care setting. Despite the success of various trials illustrating the impact of physical activity in preventing diabetes among individuals with prediabetes, it is important that this healthy behaviour be sustained beyond the intrapersonal level [15]. Changes are thus needed at other spheres of influences to achieve this. Finally, for those with prediabetes, the first contact point in the healthcare landscape is usually the primary care setting [19]. As these patients often have no obvious signs or symptoms, as well as lack understanding on the condition, it will be critical to intervene in the primary care setting to prevent diabetes for this group [20]. To achieve this, it will be useful to understand their needs on physical activity. Most studies however either focused on facilitators or barriers to physical activity, but not both [10, 12, 13]. Therefore, to enable policymakers and practitioners to plan more effective programmes for this group, it is important to understand both the barriers and facilitators of physical activity.

Similar to other Asian countries such as India and China, prediabetes and diabetes are also common in Singapore. By 2030, 1 in 4 Singapore residents will have prediabetes [21]. Likewise, the prevalence of diabetes in individuals aged 20 to 79 years will increase from 12.8% in 2014 to 22.7% in 2035 [21]. There are also huge economic costs among working individuals affected with diabetes in Singapore, where it has been estimated to increase from USD787 million in 2010, to USD1,867 million in 2050 [22]. As such, the objectives of the study were to assess the correlates of meeting physical activity recommendation, and to explore the facilitators and barriers related to the behaviour among primary care patients with prediabetes in Singapore, using the SEM. The study results would be useful for planning more tailored physical activity interventions among those with prediabetes in Asia Pacific.

## Methods

### Study design

The mixed methods approach was adopted, consisting of a cross-sectional survey (quantitative phase) and then in-depth interviews (IDIs) (qualitative phase). The explanatory sequential design was applied [23]. The first phase involved the collection and analysis of quantitative data where participants were categorised according to whether they met the physical activity recommendation. The subsequent phase involving qualitative interviews built on the previous quantitative results to explore facilitators and barriers for the behaviour in more detail. We have previously conducted studies using the mixed methods approach on dietary behaviour [24] as well as health communication and education needs [25] among primary care patients with prediabetes in Singapore. As this approach was found to be useful, we would be adopting the same methodology for this study. Since the details have been described previously, a summary would only be provided in the subsequent sub-sections.

### Quantitative phase

This occurred in 8 out of 20 polyclinics in Singapore between July 2017 and January 2018. Polyclinics are public healthcare institutions where primary care doctors and other healthcare professionals such as dietitians and nurses deliver medical care. Approximately half of the patient population with chronic illnesses in Singapore are treated in this setting [26]. During the time when the study was conducted, the polyclinics were managed by 2 public healthcare organisations, SingHealth and National Healthcare Group (NHG). There were 9 polyclinics under NHG and 11 polyclinics under SingHealth. Upon invitation, NHG agreed to participate. Eight out of the 9 polyclinics participated while the other one declined due to operational constraints.

For the cross-sectional survey, the inclusion criteria were (i) community-dwelling patients with existing prediabetes who were Singapore citizens or Singapore Permanent Residents, aged 21 to 79 years, (ii) diagnosis verified by oral glucose tolerance test (OGTT) and diagnosis code, and (iii) currently following up at any one of the 8 polyclinics. Individuals who had converted back to normoglycemia or progressed to diabetes based on the last diagnosis code and laboratory test were excluded. The polyclinic headquarter database formed the sampling frame, where patients with a diagnosis code of “impaired fasting glycaemia (IFG)” or “impaired glucose tolerance (IGT)” without “diabetes mellitus” were identified. We adopted the definitions of IFG and IGT from the World Health Organisation (WHO) [27]. Time location sampling was conducted at the polyclinic level. This meant that participants were recruited from the 8 different polyclinic venues at different times of the day, throughout the operating hours on weekdays and Saturdays (closed on Sundays). Individuals who want to undergo any testing or see a healthcare professional in polyclinics have to make prior appointments. Based on a pre-determined sampling frame, field recruiters would wait at the specific polyclinic and invite patients who turned up for their appointments to participate. These appointments need not necessary be for prediabetes follow-up, and could be for any reason.

### Sample size calculation

With precision of 5% and confidence interval (CI) of 95%, assuming a prevalence of 60% who reported physical inactivity, the estimated minimum sample size would be 283 [28]. We aimed to recruit 400 participants for the survey after accounting for a 70% response rate [24].

### Survey questionnaire

The questionnaire was self-administered, and the recruiter was nearby to provide clarification if required. Depending on the participant’s language preference, the questionnaire was available in English, Mandarin or Malay. We took actions to minimise social desirability biases, such as the use of non-judgemental questions, use of frequency-based rather than leading questions, and stressing the anonymity of their responses.

### Assessment of dependent variable

Physical activity was assessed using the global physical activity questionnaire (GPAQ) which has been validated in the local population [29]. Meeting the physical activity recommendation was defined as fulfilling at least one of the criteria of the WHO’s global physical activity recommendation [30]: (i) 150 min of moderate-intensity physical activity in a typical week, or (ii) 75 min of vigorous-intensity physical activity in a typical week, or (iii) an equivalent combination of moderate and vigorous-intensity physical activity achieving at least 600 metabolic equivalent (MET) minutes in a typical week. MET is the ratio of a person’s working metabolic rate relative to the resting metabolic rate. One MET is defined as the energy cost of sitting quietly, and it is equivalent to a caloric consumption of 1 kcal/kg/hour. MET values were calculated by multiplying weekly vigorous-intensity activity in minutes by 8 and weekly moderate-intensity activity in minutes by 4 [31].

### Assessment of independent variables

Before the study, we reviewed the literature to select the possible factors that could contribute to differences in meeting the physical activity recommendation. These could be broadly categorised into 3 main groups, (i) sociodemographic factors such as sex, ethnicity, marital status, education level, housing type, current work status and age, (ii) medical history, as well as (iii) sedentary behaviour and the availability of family members/friends for companionship during exercise. Sedentary behaviour was assessed by using a self-reported question from the National Health Survey Singapore [32], “How much time do you usually spend sitting or reclining on a typical day?” We specified in the questionnaire that this included the time spent on sitting or reclining at work, at home, getting to and from places, or with friends, including time spent [sitting at a desk, sitting with friends, travelling in car, bus, train, reading, playing cards or watching television], but excluding the time spent sleeping.

### Statistical analysis

The prevalence of participants meeting the physical activity recommendation was obtained. Bivariate analysis between each independent variable and meeting the recommendation was conducted. The association between each variable and meeting the recommendation was evaluated employing mixed effects Poisson regression model accounting for clustering by polyclinic venue. The crude prevalence ratio (PR) and 95% CI was obtained. Poisson was preferred here, rather than logistic regression, as more than 10% of the study population met the recommendation [33]. Thereafter, those with crude PR of *P* < 0.10 were selected for multivariable analysis to identify the independent factors. A backward stepwise approach was then performed to obtain the adjusted PR (aPR) and 95% CI, where only variables with *P* ≤ 0.05 were included in the final model. The study sample was also compared with the polyclinic headquarter database consisting of all the patients in NHG polyclinics with prediabetes pertaining to sex, marital status and age. We performed all statistical analyses using STATA version 15.0 [28].

### Qualitative phase

Forty-eight out of 433 who participated in the first phase underwent IDIs from September 2017 to April 2018 at the National University of Singapore. Prior to the interviews, we asked for their willingness to participate in the IDIs in the survey questionnaire. We used the maximum variation sampling strategy to select a purposive sample from diverse backgrounds, based on sex and whether they reported meeting the recommendation. We created 4 matrices based on these criteria (i. female who reported meeting the recommendation, ii. female who reported not meeting the recommendation, iii. male who reported meeting the recommendation, and iv. male who reported not meeting the recommendation). We contacted participants who had indicated their interest earlier and also fulfilled the criteria for each of these matrices. Using the SEM as a framework, we conducted the interviews employing a topic guide based on whether they reported meeting the recommendation (Additional file 1). This was available in English, Malay and Mandarin. The guide was pilot tested before study commencement to allow smooth flow and coherence. The interviewers who were the first and seventh authors conducted the interviews according to the participant’s preferred language. This ranged from 30 min to an hour. The interviews were audio recorded with consent. We reached data saturation.

### Qualitative data analysis

The interviews were transcribed verbatim and their accuracy was verified against the recordings. Interviews conducted in Malay and Mandarin were translated into English before transcription. These were then imported into NVivo 11.0 and coded line-by-line. Thematic data analysis was carried out, guided by the 6-step procedure from Braun and Clarke 2006 [34]. This involved multiple reading of the transcripts to get familiarised with the data. The initial codes were subsequently generated by the first and seventh authors independently before coming together to establish inter-coder reliability. This was achieved through discussing and resolving discrepancies in coding through discussion involving all team members. The codebook was continuously refined with additional codes emerging during the process. This occurred iteratively until inter-coder reliability was achieved at the 10th transcript. The finalised codebook was used to code the remaining transcripts. The codes were then categorised and condensed into preliminary subthemes and themes by the same 2 authors independently. Any discrepancy was again resolved by group consensus.

### Ethics approval and participant consent

The study was approved by the National Healthcare Group Domain Specific Review Board (approval certificate number 2016/01358) in accordance with the Declaration of Helsinki. Informed written consent was obtained from all the participants.

## Results

### Quantitative phase results

Out of 948 whom we approached, a total of 648 responded. The participation rate was 66.8% where 433 out of the 648 agreed to take part. Non-participants did not differ significantly from participants in terms of sex, age and ethnicity. “Busy” and “not interested” were the main reasons cited for non-participation. Participants mirrored the total NHG Polyclinic patient pool with prediabetes in sex, marital status and age (results not shown) [35, 36]. Table 1 showed the survey participant characteristics. The prevalence of participants who reported meeting the physical activity recommendation was 65.8%. The mean sitting or reclining hours on a typical day was 4.2 (standard deviation: 2.9). Of the 148 participants who did not fulfil the physical activity recommendation, 39 (26.4%) sat or reclined at least 8 hr per day (high level of sedentary behaviour, results not shown in Table 1). In addition, the prevalence of participants with osteoarthritis was 14.3% (results not shown in Table 1). Of note, there was no statistical difference in current work status among those who met the recommendation versus those who did not.

**Table 1:** Comparison of sociodemographic characteristics, medical history and behaviour for those meeting and not meeting the physical activity recommendation

Characteristic	Did not meet the recommendation (*n* = 148)	Met the recommendation (*n* = 285)	P value^+^
Sociodemographic characteristics			
Sex			
Female	84 (56.8)	127 (44.6)	0.02
Male	64 (43.2)	158 (55.4)	
Ethnicity			
Chinese	113 (76.4)	236 (82.8)	0.26
Malay	19 (12.8)	31 (10.9)	
Indian	13 (8.8)	16 (5.6)	
Others	3 (2.0)	2 (0.7)	
Marital status			
Single	26 (17.6)	34 (11.9)	0.11
Married	122 (82.4)	251 (88.1)	
Highest education level			
No formal education	11 (7.4)	7 (2.5)	0.001
Primary	46 (31.1)	69 (24.2)	
Secondary	65 (43.9)	116 (40.7)	
Post-secondary	26 (17.6)	93 (32.6)	
Housing type*			
1–3 room public housing	36 (24.7)	45 (15.8)	0.05
4–5 room public housing	86 (58.9)	174 (61.0)	
Executive flat/private property	24 (16.4)	66 (23.2)	
Current work status			
Currently working	83 (56.1)	162 (56.8)	0.88
Not working	65 (43.9)	123 (43.2)	
Age in years, mean (SD)	62.6 (8.9)	61.7 (8.3)	0.27
Medical history			
Type of prediabetes			
Impaired fasting glycaemia	80 (54.1)	151 (53.0)	0.83
Impaired glucose tolerance	68 (45.9)	134 (47.0)	
History of osteoarthritis			
No	116 (78.4)	255 (89.5)	0.002
Yes	32 (21.6)	30 (10.5)	
Years with prediabetes, mean (SD)	1.9 (2.1)	2.1 (2.3)	0.28
Behaviour			
Having family members/friends to exercise with			
No	134 (90.5)	166 (58.2)	< 0.001
Yes	14 (9.5)	119 (41.8)	
Number of hours spent sitting or reclining daily, mean (SD)*	4.7 (3.7)	3.9 (2.4)	0.006

All figures in the table referred to frequency (column percentage) unless otherwise indicated

* Contained missing numbers (housing type, 2; and number of hours spent sitting or reclining daily, 1)

^**+**^ The *p*-values were computed using χ2 test or Fisher Exact test (whichever appropriate) for categorical variables and two-sample t-test for continuous variables

Table 2 showed the crude and adjusted PR of factors associated with meeting the physical activity recommendation. Compared with females, males had a higher prevalence of meeting the recommendation (PR 1.18; 95%CI: 1.07–1.31). Compared with those staying in 1–3 room public housing (affordable flats, with different number of rooms for each housing type, developed and administered by the government; 80% of Singapore residents live in these flats), those staying in 4–5 room public housing (PR 1.20; 95%CI: 1.09–1.33) and those staying in executive flat/private property (PR 1.32; 95%CI: 1.10–1.59) had a higher prevalence of meeting the recommendation. Furthermore, there was a positive dose-response relationship as the *p* value for the trend was 0.02. The prevalence of meeting the recommendation was lower in those with osteoarthritis than those who did not (PR 0.70; 95%CI: 0.53–0.94). Compared with participants who did not have companionship of family members/friends during exercise, the prevalence of meeting the recommendation was higher among those who did (PR 1.62; 95%CI: 1.41–1.86). The prevalence of meeting the recommendation also decreased with the number of hours spent sitting or reclining daily (PR 0.97; 95%CI: 0.95–0.98).

**Table 2:** Crude and adjusted prevalence ratio (PR) of sociodemographic characteristics, medical history and behaviour associated with meeting the physical activity recommendation

Characteristic	Crude PR (95% CI)	Adjusted PR^+^ (95% CI)
Sociodemographic characteristics		
Sex		
Female	Referent	
Male	1.18 (1.07–1.31)	1.21 (1.09–1.34) ^#^
Ethnicity		
Chinese	Referent	
Malay	0.92 (0.77–1.10)	0.99 (0.82–1.18)
Indian	0.82 (0.61–1.08)	0.85 (0.71–1.02)
Others	0.59 (0.24–1.45)	0.77 (0.35–1.67)
Marital status		
Single	Referent	
Married	1.19 (0.93–1.52)	0.99 (0.78–1.25)
Highest education level		
No formal education	Referent	
Primary	1.54 (0.66–3.59)	1.33 (0.62–2.84)
Secondary	1.65 (0.72–3.80)	1.42 (0.67–3.01)
Post-secondary	2.01 (0.87–4.62)	1.80 (0.86–3.77)
Housing type*^		
1–3 room public housing	Referent	
4–5 room public housing	1.20 (1.09–1.33)	1.19 (1.07–1.31) ^#^
Executive flat/private property	1.32 (1.10–1.59)	1.26 (1.06–1.50) ^#^
Current work status		
Currently working	Referent	
Not working	0.99 (0.91–1.08)	0.96 (0.85–1.08)
Age in years	1.00 (0.99–1.00)	0.99 (0.98–1.00)
Medical history		
Type of prediabetes		
Impaired fasting glycaemia	Referent	
Impaired glucose tolerance	1.01 (0.94–1.09)	1.01 (0.94–1.08)
History of osteoarthritis		
No	Referent	
Yes	0.70 (0.53–0.94)	0.75 (0.59–0.96) ^#^
Years with prediabetes	1.02 (0.99–1.04)	1.01 (0.99–1.03)
Behaviour		
Having family members/friends to exercise with		
No	Referent	
Yes	1.62 (1.41–1.86)	1.57 (1.38–1.78) ^#^
Number of hours spent sitting or reclining daily*	0.97 (0.95–0.98)	0.96 (0.94–0.98) ^#^

* Contained missing numbers (housing type, 2; and number of hours spent sitting or reclining daily, 1)

^**+**^ The aPR of the variables that were not significant at the 5% level was obtained by incorporating that particular variable in the final multivariable model

^#^ These variables were significant at the 5% level and were included in the final multivariable model using the backward stepwise approach

^ The *p* value for trend was 0.02

On multivariable analysis, the prevalence of meeting the recommendation was positively associated with being male (aPR 1.21, 95%CI 1.09–1.34), living in 4–5 room public housing (aPR 1.19, 95%CI 1.07–1.31), living in executive flat/private housing (aPR 1.26, 95%CI 1.06–1.50), having the companionship of family members/friends during exercise (aPR 1.57, 95%CI 1.38–1.78). In contrast, the prevalence was negatively associated with having a history of osteoarthritis (aPR 0.75, 95%CI 0.59–0.96) and time spent sitting or reclining daily (aPR 0.96, 95%CI 0.94–0.98).

### Qualitative phase participant characteristics

Table 3 showed the IDI participant characteristics.

**Table 3:** Participant characteristics for the in-depth interviews

Characteristic	*N* = 48
Sociodemographic characteristics	
Sex	
Female	24 (50.0)
Male	24 (50.0)
Ethnicity	
Chinese	37 (77.1)
Malay	6 (12.5)
Indian	5 (10.4)
Age in years, mean (SD)	59.8 (9.1)
Behaviour	
Physical activity recommendation	
Meeting	24 (50.0)
Not meeting	24 (50.0)

All figures in the table referred to frequency (column percentage) unless otherwise indicated

#### Qualitative phase results: facilitators for meeting the recommendation

**Fig. 1:**
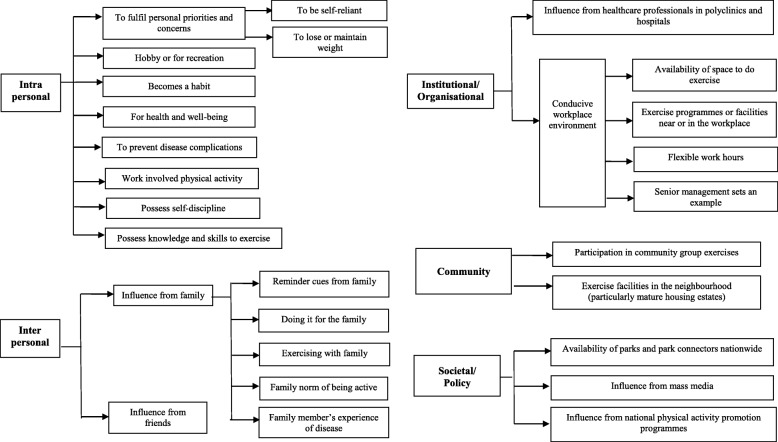
Themes and subthemes pertaining to reasons for meeting the physical activity recommendation

Figure 1 showed the overview of the themes and subthemes pertaining to facilitators for those who reported meeting the recommendation. At the intrapersonal level, participants met the recommendation for reasons pertaining to health and well-being, *“So, when you go outside to exercise, you feel the sunshine, you breathe in the fresh air, your body will then be good. It is for our well-being.”* (*AMK 070, 59 years old Chinese female*). Participants also wanted to prevent disease complications, particularly diabetes, *“Oh because of this prediabetes problem. That’s why I thought better to do some exercise and increase my activity level. I don’t want to get diabetes.”* (*AMK 007, 68 years old Chinese female*).

Other participants engaged in physical activity as a hobby or for recreation. For some, it had become a habit, *“Once exercise becomes a habit, I don’t think you want to give it up. It becomes a part of you. One day if I miss out on the cycling, I will feel uncomfortable.”* (*YIS 015, 51 years old Chinese male*). For others, it was to fulfil their personal priorities and concerns, including being self-reliant, *“Yes, the aim is to rely on yourself. I keep thinking to myself if I don’t be active and take charge of my own health, nobody can.”* (*CCK 001, 56 years old Chinese female*).

The main recurrent themes were observed at the interpersonal level where family/peer influence played an important role in facilitating the recommendation. This could be in the form of receiving reminder cues from family to exercise, *“My son encourages me by asking me like every other day, did you exercise today? Oh, you didn’t, you must exercise!”* (*AMK 078, 56 years old Chinese female*). It could also be through motivation from exercising with peers, *“I do exercise with my friends, one or two days a week, play badminton, play games or jog.”* (*WDL 056, 44 years old Indian male*).

For some, it was the family norm to be active, *“So, my whole family all very active. Even my mother until today, ninety-two already, still physically active. Until now, all my brothers and sisters, they are all five years younger than me, but they also have their form of exercise, it’s in our family culture.”* (*AMK 054, 67 years old Chinese male*). For others, they were doing it for the family, *“I also feel that I need to keep a healthy body because I want to depend on myself. I don’t want to burden my family, that is why I exercise regularly to keep healthy for my family.”* (*YIS 008, 69 years old Malay male*).

At the institutional/organisational level, participants met the recommendation because of influence from healthcare professionals in polyclinics and hospitals, *“Yes, the polyclinic doctor advises me to do some exercise like brisk walk, so I have to do it. I mean what the doctor says is good for me right? So, I follow …*” (*WDL 019, 65 years old Malay male*). For others, a conducive work environment facilitated meeting the recommendation. These participants were able to access exercise programmes or facilities near or in their workplace. Yet others felt that their flexible work hours were useful, *“Yes, my workplace allows me to work flexible that’s why it is more convenient for me to arrange my schedule such that I can fit in my exercise regimen.”* (*AMK 100, 51 years old Chinese male*).

At the community level, participants took part in community group exercises, *“I do have an exercise group in the neighbourhood that I live in, we exercise together, motivate each other, and remind each other to do exercise.”* (*TPY 014, 65 years old Chinese male*). In addition, some participants would utilise the exercise facilities in their neighbourhood, particularly for mature housing estates, *“Yes, at the neighbourhood where I stay which is considered a mature area, there is the gym and the swimming pool, so it is convenient for me to use these facilities.”* (*CCK 001, 56 years old Chinese female*).

At the societal/policy level, the availability of parks and park connectors nationwide facilitated meeting the recommendation for some participants, *“...nowadays they have a lot those parks being connected to one other, including the one at my place too, so it is convenient to exercise there.”* (*TPY 019, 54 years old Chinese female*). Park connectors are walking/running/cycling paths that connect various parks and other green spaces in Singapore.

For others, it was due to the influence of mass media, *“The TV, especially those programmes talking about health, they will show you how to take care of your health, how to be active.”* (*WDL 019, 65 years old Malay male*). Some attributed it to the national physical activity promotion programmes, “*… the government is also encouraging, they give the Active SG credit for a year for all citizens which we can use to book table tennis, entrance to swimming pool, so I make use of it.”* (*AMK 075, 56 years old Chinese female*). The Active SG is a national programme where Singapore residents are provided with SGD$100 credit online to sign up for physical activities or to pay for usage of public physical activity facilities.

#### Qualitative phase results: barriers in those not meeting the recommendation

**Fig. 2:**
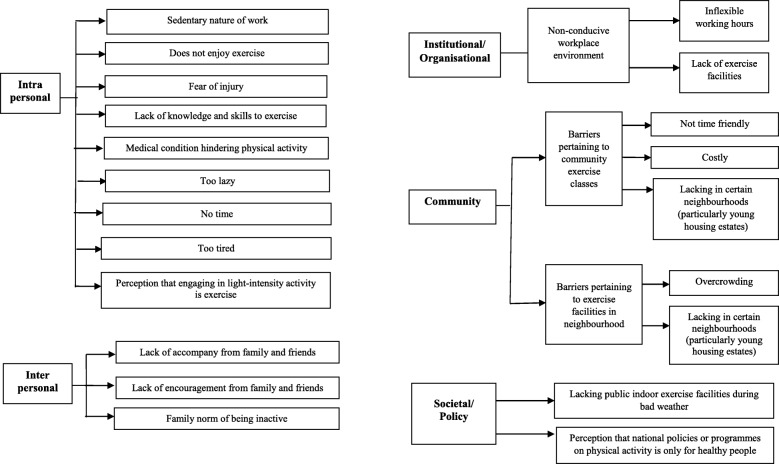
Themes and subthemes pertaining to reasons for not meeting the physical activity recommendation

Figure 2 showed the overview of the themes and subthemes pertaining to barriers for those who reported not meeting the recommendation. At the intrapersonal level, many participants did not meet the recommendation because they had “no time”. Their commitments differed by sex. Most female non-doers cited the various life roles particularly family obligations they had to fulfil which they regarded as more important than physical activity. These included their roles as wives, mothers, daughters, and in some cases, caregivers, *“from Monday to Friday I’m working .... then Saturday and weekend I need to run errands for my children, my husband, and on top of that there is the housework. I also need to spend some time to visit my parents. Time is very important to me, I have so many duties and roles to fulfil, my first priority is always my family.”* (*YIS 002, 57 years old Malay female*). This contrasted with male non-doers, who mostly cited work as the reason for “no time”.

For others, they either felt too lazy to engage in physical activity or too tired to do so. Some non-doers lacked the knowledge and skills to exercise, *“I don’t know much about exercises, like how to exercise properly, how to protect my knees when exercising, which exercises are suitable for me and my medical condition.”* (*HOU 041, 46 years old Indian female*). There were others who erroneously perceived that light-intensity activity was exercise, *“So, doing housework is enough physical activity for me, walking in the house is exercise.”* (*AMK 005, 63 years old Chinese female*).

For others, their medical conditions hindered physical activity engagement, particularly if they had osteoarthritis, “*… at one time my knee gives the cricking sound when I do the Zumba dance. And at another time it was inflamed, in fact a few times. And doctor say it’s wear and tear, so I decide to cut down on exercise.”* (*AMK 097, 58 years old Chinese female*). Participants also feared sustaining injury during exercise, *“Once an elderly falls down...it is very bad...becomes a burden to the family … that’s why I rather don’t do exercise, don’t move about so much … I don’t want to injure myself.”* (*WDL 048, 77 years old Chinese male*).

The main recurrent themes were observed at the interpersonal level where family/peer influence played an important role in not meeting the recommendation. Participants perceived the lack of companionship from their family and friends, during exercise, as a barrier, *“I find that indoor gym work is a bit boring, unless there is someone to accompany me like my family or my friends. Then it is ok, we can talk and exercise at the same time. If not, one person alone I won’t go.”* (*AMK 017, 57 years old Chinese female*).

For some, the lack of encouragement from their family or friends was an important barrier, *“I have no one to motivate me. No one in the family or my friend encourages me to exercise.”* (*YIS 005, 57 years old Malay female*). For others, it was the family norm to be inactive, *“No, my family also never do exercise. It is not our family culture to do any exercise, has been like that since the children are young.”* (*HOU 035, 65 years old Chinese female*).

At the institutional/organisational level, some participants cited non-conducive work environments, such as lack of exercise facilities, as a barrier, *“There is no gym for us to use at the workplace.”* (*WDL 012, 50 years old Chinese male*).

At the community level, participants experienced barriers pertaining to community exercise classes, such as high cost, *“For example, my neighbourhood there, they got this yoga class in a mall, but the monthly subscription is SGD$110, that is costly for me, since most of us are semi-retired at this age. The Active SG credit cannot be used to pay for this as the class is not part of their recognised list.”* (*TPY 022, 56 years old Chinese male*). The lack of such classes in certain neighbourhoods, particularly for young housing estates, was cited as another barrier. In addition, participants faced barriers relating to exercise facilities in the neighbourhood, such as overcrowding, *“My neighbourhood has outdoor fitness equipment, but I don’t really like because it is crowded. The equipment there is always occupied, and you have to wait, so forget about it.”* (*AMK 017, 57 years old Chinese female*).

At the societal/policy level, there was the perception that national policies on physical activity programmes was only applicable to healthy people, *“The on-going government campaigns do encourage us to exercise. But these are targeted at people who can exercise. They don’t have the knee pain or arthritis, they are okay. For people like us who have the knee pain or some other medical conditions, I don’t see any specific campaigns for us.”* (*AMK 005, 63 years old Chinese female*). The lack of public indoor exercise facilities that could be used during bad weather also emerged as a barrier.

## Discussion

Two in 3 primary care patients with prediabetes met the WHO’s physical activity recommendation. This was more prevalent among those who were male, those who stayed in 4 to 5-room public housing, those who stayed in executive flat/private property and those with family members/friends to exercise with. In contrast, fulfilling the recommendation was less prevalent among those with osteoarthritis and those who spent more hours sitting or reclining daily. Reasons existed at different levels for facilitators and barriers related to physical activity. The recurrent themes for not meeting the recommendation included lacking companionship from family members/friends, medical conditions hindering physical activity (particularly osteoarthritis), lacking knowledge/skills to exercise properly, “no time” to exercise and barriers pertaining to exercise facilities in the neighbourhood. The recurrent themes for meeting the recommendation included family/peer influence, health/well-being concerns and education by healthcare professionals.

The correlates from the quantitative phase were generally supported by barriers and facilitators from the qualitative phase, hence there was data triangulation. For example, pertaining to facilitators of physical activity, the correlate of having family members/friends to exercise with from the quantitative component concurred with the recurrent theme of family/peer influence from the qualitative component. Similarly, for barriers to physical activity, the correlate of having a history of osteoarthritis from the quantitative phase concurred with the recurrent theme of medical conditions hindering physical activity (particularly osteoarthritis) from the qualitative phase.

The proportion of those who met the recommendation (65.8%) in our study population was slightly higher compared to other studies where the prevalence ranged from 27.3 to 57.3% among those with prediabetes [10–13, 37, 38]. Consistent with other findings, the prevalence of having sufficient physical activity was more common among males [12, 13]. The IDIs suggested that the various roles that females undertook in society, compared to males, was likely a key factor. This was also consistent with other studies where women typically reported greater barriers, such as household and caregiving responsibilities, which took precedence over physical activity [39]. Interestingly, housing status showed a dose-response relationship with meeting the recommendation. This was similar to another study in South Korea where Lee et al. reported that among metabolic syndrome patients at risk of diabetes, physical activity adherence was lowest in the first (28.3%) quartile of socioeconomic status and highest in the fourth quartile (43.8%) [40]. It was not clear from our IDIs how housing status contributed to this difference. A future study would lend valuable insights into socioeconomic status as a possible determinant for physical activity and the causal pathways at work.

Meeting the recommendation was negatively associated with sedentary time spent reclining or sitting. Sedentary behaviour is associated with an increased risk of diabetes, independent of physical inactivity [41]. According to a meta-analysis, greater sedentary time was associated with a significantly increased risk of diabetes even after adjustment for physical activity (pooled relative risk 2.47; 95%CI 1.49–3.95) [42]. Our results revealed that among those who were physically inactive, slightly more than a quarter also had sedentary behaviour of at least 8 hr. This group would be at the greatest risk for diabetes. Other than promoting physical activity, special attention should also be directed towards decreasing sedentary time among patients with prediabetes. This would further reduce their risk of diabetes.

Meeting the recommendation was positively associated with companionship of family members/friends during exercise. This was also a major recurrent theme in our IDIs. Consistent with other studies, “needing family or friends to exercise with” was associated with physical activity engagement among older adults at risk of diabetes (adjusted odds ratio, aOR 2.7; 95%CI 1.5–4.9) [12]. Family/friends have both, direct and indirect positive influence from our IDI results. The direct influence would come from exercising together with the participants; alternatively, the influence may also be indirect, by providing encouragement or reminders. This is not surprising given that the family is often regarded as the basic societal unit in Asia [43]. Therefore, leveraging family/peer support is important to promote physical activity in an Asian population. On the other hand, family/peer could also pose a negative influence on physical activity. To address this, instead of placing sole attention on patients with prediabetes, their family members and peers could be engaged as agents of change. To begin, health promotion messages on physical activity, targeting those with prediabetes, could incorporate the theme of “exercise with your family or friend”. Other settings such as the primary care setting, workplaces and the community could also be utilised to implement more family or peer-based physical activity promotion activities.

Meeting the recommendation was negatively associated with having osteoarthritis. This was also highlighted in our IDIs. Another study reported this association in the United States where arthritis was associated with insufficient physical activity among adults (aOR 1.6; 95%CI 1.1–2.2) [13]. During the IDIs, participants shared that they lacked the knowledge and skills to exercise. This was particularly so among those with osteoarthritis as they did not know which exercises were appropriate for their condition. Among the facilitators to physical activity, influence from healthcare professionals in polyclinics and hospitals was listed at the organisational/institutional level of the SEM. Therefore, in the primary care setting, physical activity prescription and coaching could be introduced to address this lack of exercise knowledge and skills. Given that osteoarthritis was one of the five leading causes of disability in Singapore [44] and was present in close to one-fifth of our participants, healthcare professionals, particularly doctors, could specifically look out for arthritis-related functional limitations when assessing for prediabetes progression in their patients. In addition, at the societal/policy level, there was the perception that national policies or programmes regarding physical activity were only applicable to healthy people. It is important to remember that individuals with prediabetes are not a homogenous population; a nuanced approach that addresses various needs and preferences will be required. Accordingly, national health promotion messages and programmes on physical activity should also promote non–weight-bearing exercises such as stationary cycling, armchair exercises, and aquatic exercises as alternatives to walking or jogging for the population with prediabetes.

Research into physical activity has always focused on intrapersonal and interpersonal factors, partly due to the difficulties in examining structural and social influences [15]. Our results showed that physical activity promotion should make its way into the community. Aside from the intrapersonal and interpersonal levels, several barriers and facilitators relating to physical activity existed at the organisational, community and societal/policy levels. Being the first line contact, the effectiveness of delivering education by healthcare professionals in the primary care setting cannot be over-emphasised. However, promotion of physical activity and reduction of sedentary behaviour would need to go beyond the healthcare system to the national and societal level. We have found how environmental modification measures taken by the government, such as the park connector network, as well as the Active SG credit national programme, have served as facilitators to physical activity in the IDI results. Other organisations and settings, such as workplaces, the neighbourhood and the community at large, should also be effectively engaged to promote behavioural change. As a start, other than initiating conversations on physical activity, polyclinic healthcare professionals should also direct patients with prediabetes towards appropriate community resources. In addition, practitioners and policy makers must continue to address the social, cultural and physical barriers to physical activity, in tandem with national policy refinements, to promote physical activity. An example would be to increase the types of physical activity offered by community centres, particularly in the young housing estates, as well as to increase the list of programmes or classes that could be payable with the Active SG credit.

### Limitations and strengths

There were some limitations. Firstly, we could not exclude social desirability bias since there was sole dependence on self-reported data. Nevertheless, we have described in the Methods section the steps which have been taken to reduce this bias. Secondly, we cannot infer causal relationships from a cross-sectional study (first phase). Thirdly, we did not show the transcript to the participants during the qualitative phase to confirm whether their responses had been accurately documented. This was however mitigated by constantly paraphrasing and “checking back” with the participants to ascertain the accuracy of their responses. There were notable strengths despite the limitations. The GPAQ component of the survey has been validated in the local population. This was one of the very few studies to utilise a mixed methods approach in understanding the correlates, barriers and facilitators pertaining to meeting the physical activity recommendation among primary care patients with prediabetes. This approach facilitated triangulation since not only were some of the correlates in the quantitative analysis recurrent themes in the qualitative analysis, the IDIs also enabled better understanding of these correlates. We reached data saturation for the qualitative analysis. Although our study sample was not generalisable to all individuals with prediabetes in Singapore, it largely reflected the total NHG Polyclinic patient pool with prediabetes.

## Conclusions

Much more remains to be done to promote physical activity among primary care patients with prediabetes in Singapore. Participants reported facilitators and barriers to physical activity at different levels of the SEM. Apart from the individual and interpersonal levels, practitioners and policy makers need to work together to address the organisational, community and policy barriers to physical activity.

## Supplementary information

**Additional file 1.** Topic guide used for in-depth interviews.

## Data Availability

The data used to support the findings of this study are included within the article, which are available from the corresponding author upon request.

## Abbreviations

aOR: adjusted odds ratios
aPR: adjusted prevalence ratios
CI: confidence interval
GPAQ: Global Physical Activity Questionnaire
IDIs: in-depth interviews
IFG: impaired fasting glycaemia
IGT: impaired glucose tolerance
OGTT: oral glucose tolerance test
PR: prevalence ratio
SEM: socio-ecological model
WHO: World Health Organisation

## References

[1] Bansal N (2015). Prediabetes diagnosis and treatment: a review. World J Diabetes.

[2] Tabák AG, Herder C, Rathmann W, Brunner EJ, Kivimäki M (2012). Prediabetes: a high-risk state for diabetes development. Lancet..

[3] Lim R, Chen C, Naidoo N (2015). Anthropometrics indices of obesity, and all-cause and cardiovascular disease-related mortality, in an Asian cohort with type 2 diabetes mellitus. Diabetes Metab.

[4] Allender S, Foster C, Hutchinson L, Arambepola C (2008). Quantification of urbanization in relation to chronic diseases in developing countries: a systematic review. J Urban Health.

[5] Deepa M, Grace M, Binukumar B (2015). High burden of prediabetes and diabetes in three large cities in South Asia: the center for cArdio-metabolic risk reduction in South Asia (CARRS) study. Diabetes Res Clin Pract.

[6] Hootman JM (2009). 2008 physical activity guidelines for Americans: an opportunity for athletic trainers. J Athl Train.

[7] Colberg SR, Sigal RJ, Yardley JE (2016). Physical activity/exercise and diabetes: a position statement of the American Diabetes Association. Diabetes Care.

[8] Roberts CK, Hevener AL, Barnard RJ (2013). Metabolic syndrome and insulin resistance: underlying causes and modification by exercise training. Compr Physiol.

[9] Hallsworth K, Fattakhova G, Hollingsworth KG (2011). Resistance exercise reduces liver fat and its mediators in non-alcoholic fatty liver disease independent of weight loss. Gut..

[10] Taylor LM, Spence JC, Raine K, Sharma AM, Plotnikoff RC (2011). Self-reported physical activity preferences in individuals with prediabetes. Phys Sportsmed.

[11] Vähäsarja K, Salmela S, Villberg J (2014). Perceived sufficiency of physical activity levels among adults at high risk of type 2 diabetes: the FIN-D2D study. Int J Behav Med.

[12] Han BH, Sadarangani T, Wyatt LC (2016). Correlates of physical activity among middle-aged and older Korean Americans at risk for diabetes. J Nurs Scholarsh.

[13] Strauss SM, McCarthy M (2017). Arthritis-related limitations predict insufficient physical activity in adults with Prediabetes identified in the NHANES 2011-2014. Diab Educ.

[14] Taylor LM, Raine KD, Plotnikoff RC, Vallance JK, Sharma AM, Spence JC (2016). Understanding physical activity in individuals with prediabetes: an application of social cognitive theory. Psychol Health Med.

[15] Hill JO, Galloway JM, Goley A (2013). Scientific statement: Socioecological determinants of prediabetes and type 2 diabetes. Diabetes Care.

[16] Thornton CM, Kerr J, Conway TL (2017). Physical activity in older adults: an ecological approach. Ann Behav Med.

[17] Elder JP, Lytle L, Sallis JF (2007). A description of the social-ecological framework used in the trial of activity for adolescent girls (TAAG). Health Educ Res.

[18] Mehtälä MA, Sääkslahti AK, Inkinen ME, Poskiparta ME (2014). A socio-ecological approach to physical activity interventions in childcare: a systematic review. Int J Behav Nutr Phys Act.

[19] Mainous AG, Tanner RJ, Baker R (2016). Prediabetes diagnosis and treatment in primary care. J Am Board Fam Med.

[20] O'Brien MJ, Moran MR, Tang JW (2016). Patient perceptions about Prediabetes and preferences for diabetes prevention. Diab Educ.

[21] Wong LY, Toh MP, Tham LW (2017). Projection of prediabetes and diabetes population size in Singapore using a dynamic Markov model. J Diab.

[22] Nanditha A, Ma RC, Ramachandran A (2016). Diabetes in Asia and the Pacific: implications for the global epidemic. Diabetes Care.

[23] Creswell JW, Clark VLP (2007). Designing and conducting mixed methods research.

[24] Lim RBT, Wee WK, For WC (2019). Correlates, facilitators and barriers of healthy eating among primary care patients with prediabetes in singapore-a mixed methods approach. Nutrients.

[25] Lim RBT, Wee WK, For WC, et al. Health education and communication needs among primary care patients with prediabetes in Singapore: a mixed methods approach. Prim Care Diab. 2019. pii: S1751–9918(19)30116–0. 10.1016/j.pcd.2019.08.008 31558372

[26] Khoo HS, Lim YW, Vrijhoef HJ (2014). Primary healthcare system and practice characteristics in Singapore. Asia Pac Fam Med.

[27] WHO, International Diabetes Foundation (2006). Definition and diagnosis of diabetes mellitus and intermediate hyperglycaemia: report of a WHO/IDF consultation.

[28] StataCorp (2017). Stata statistical software: release 15.

[29] Chu Anne, Ng Sheryl, Koh David, Müller-Riemenschneider Falk (2018). Domain-Specific Adult Sedentary Behaviour Questionnaire (ASBQ) and the GPAQ Single-Item Question: A Reliability and Validity Study in an Asian Population. International Journal of Environmental Research and Public Health.

[30] World Health Organization (2010). Global recommendations on physical activity for health.

[31] Win AM, Yen LW, Tan KH, Lim RB, Chia KS, Mueller-Riemenschneider F (2015). Patterns of physical activity and sedentary behavior in a representative sample of a multi-ethnic south-east Asian population: a cross-sectional study. BMC Public Health.

[32] National Health Survey 2010. Singapore: ministry of health; https://www.moh.gov.sg/docs/librariesprovider5/resources-statistics/reports/nhs2010%2D%2D-low-res.pdf.

[33] Coutinho LM, Scazufca M, Menezes PR (2008). Methods for estimating prevalence ratios in cross-sectional studies. Rev Saude Publica.

[34] Braun V, Clarke V (2006). Using thematic analysis in psychology. Qual Res Psychol.

[35] Toh MP, Leong HS, Lim BK (2009). Development of a diabetes registry to improve quality of care in the National Healthcare Group in Singapore. Ann Acad Med Singap.

[36] Heng BH, Sun Y, Cheah JT, Jong M (2010). The Singapore National Healthcare Group Diabetes Registry--descriptive epidemiology of type 2 diabetes mellitus. Ann Acad Med Singap.

[37] Taylor LM, Johnson ST, Vallance JK, Stadnyk J, Basualdo-Hammond C (2014). Food and physical activity behaviours of adults attending a prediabetes education class. Can J Diabetes.

[38] Zhou QP, Oh KM (2012). Comparison of lifestyle behaviors and related factors between Asian American and white adults with prediabetes. Nurs Health Sci.

[39] Cantu AG, Fleuriet JK (2008). The sociocultural context of physical activity in older Mexican American women. Hisp Health Care Int.

[40] Lee H, Kim BH (2016). Physical activity disparities by socioeconomic status among metabolic syndrome patients: the fifth Korea National Health and nutrition examination survey. J Exerc Rehabil.

[41] González K, Fuentes J, Márquez JL (2017). Physical inactivity, sedentary behavior and chronic diseases. Korean J Fam Med.

[42] Wilmot EG, Edwardson CL, Achana FA (2012). Sedentary time in adults and the association with diabetes, cardiovascular disease and death: systematic review and meta-analysis. Diabetologia..

[43] Lim K, Kayser-Jones J, Waters C, Yoo G (2007). Aging, health, and physical activity in Korean Americans. Geriatr Nurs.

[44] Leung YY, Pua YH, Thumboo J (2013). A perspective on osteoarthritis research in Singapore. Proc Singapore Healthcare.

