# Supplementary material for: Correlates, facilitators and barriers of physical activity among primary care patients with prediabetes in Singapore – a mixed methods approach
Source: BMC Public Health. 2020 Jan 2;20:1. doi: 10.1186/s12889-019-7969-5 (PMC6941241; doi:10.1186/s12889-019-7969-5)
Supplement: Supplementary file 1 — Additional file 1. Topic guide used for in-depth interviews. [file 12889_2019_7969_MOESM1_ESM.pdf]

Supplementary Material 1: Topic guide used for in-depth interviews

ICE-BREAKER

Could you introduce yourself so that we can get to know you better?

Please use Section A or B depending on the response of the participant during the survey.

Section A. Doer analysis for physical activity

From the earlier survey that was conducted, you would have fulfilled the requirements of meeting sufficient physical activity according to the national recommendation.

Probe:

- What are your motivating factors at a personal level? Personal level refers to your knowledge, beliefs, attitudes and skills. How have these factors encouraged you to exercise or to be physically active?

- What are your motivating factors at an interpersonal level? Interpersonal level refers to your family, friends, colleagues etc. How have these people encouraged you to exercise or to be physically active?

- What are your motivating factors at community level? Community level refers to any programme or efforts by your workplace, neighbourhood, or support group to encourage you to exercise or to be physically active.

- What are your motivating factors at societal level? Societal level refers to any policy or efforts by government agencies implemented at the national level to encourage you to exercise or to be physically active.

During this process of being physically active, it would be common to face various challenges such as wanting to give up or to delay your exercise regimen.

Probe:

- Have you encountered these challenges? If yes, what did you do to tackle these challenges?

Section B. Non-doer analysis for physical activity

From the earlier survey that was conducted, you are not able to meet the national recommended level of physical activity, meaning you are not exercising enough.

Probe:

- What are the difficulties that you face at a personal level? Personal level refers to your knowledge, beliefs, attitudes and skills. How have these factors prevented you from exercising enough or being physically active?

- What are the difficulties that you face at an interpersonal level? Interpersonal level refers to your family, friends, colleagues etc. How have these people discouraged you from exercising enough or being physically active?
- What are the difficulties that you face at the community level? Community level refers to any barriers that you encountered at your workplace, neighbourhood, or support group to discourage you from exercising enough or being physically active.

- What are the difficulties that you face at the societal level? Societal level refers to any policy or action by government agencies implemented at the national level to discourage you from exercising enough or being physically active.

Probe:

- During this process of becoming more physically active, it is common to start on an exercise regimen for a while and then give it up. Have you ever tried to increase your physical activity for at least once during a certain period in your life?
    - If yes, can you share with us what you have tried?
    - How long have you tried? What made you give up in the end?
    - If no, what factors have prevented you from trying at least once?

End of in-depth interview

Is there anything else that you would like to ask or tell me?

We have come to the end of the interview. We have had a fruitful discussion and all the valuable comments that were gathered during this discussion will be used to help develop a programme that is customised to the needs of patients like you. I would like to take this opportunity to thank you for your valuable contributions.

(Discussion ends here)
